# Evaluation of the Short-Term Response in Refractory Trigeminal Neuralgia Treated With CyberKnife

**DOI:** 10.7759/cureus.48401

**Published:** 2023-11-06

**Authors:** Ambar Pérez Fernández, Randy E Aquino, Celeste Niño De Guzmán, Laura Mancebo Díaz, Moisés Mera, Heyward Solarte

**Affiliations:** 1 Radiosurgery, Oncoserv, Santo Domingo, DOM; 2 Neuroanatomy, Universidad Autónoma de Santo Domingo (UASD), Santo Domingo, DOM; 3 Medicine, Oncoserv, Santo Domingo, DOM; 4 Radiation Oncology, Oncoserve, Santo Domingo, DOM

**Keywords:** trigeminal neuralgia, short-term response, radiosurgery, pain relief, cyberknife®

## Abstract

Objectives

Stereotactic radiosurgery combined with pharmacological treatment is currently one of the most acceptable alternatives for the treatment of trigeminal neuralgia (TN). Our primary endpoint was to report the short-term response (one month) outcomes of 10 patients with refractory TN after CyberKnife^®^ (CK) radiosurgery; secondary endpoints were to assess early side effects and complications.

Methods

Ten female patients with TN refractory to pharmacological and/or non-pharmacological treatment received a single dose of 90 Gy with CyberKnife^®^ radiosurgery. Clinical and demographic characteristics were obtained from medical records. The visual analog scale (VAS) was used to assess facial pain before as well as seven, 15, and 30 days after treatment. Friedman test was performed to evaluate pain relief in treated patients.

Results

All patients responded well to the CK and experienced initial adequate pain relief during the first 30 days (p<0.001). No significant differences (p=0.661) were found between six patients who received CK radiosurgery as the first treatment option and those who underwent other non-pharmacological treatments. One patient was re-irradiated with 75 Gy. Transient facial paresthesia was observed in 3/10 patients without any other complications.

Conclusion

High-tech CK treatment is safe, non-invasive, fast, with minimal side effects, and effective in achieving short-term pain relief in patients with refractory TN, even in those with multiple previous interventions. Given these results, we recommend evaluating CK radiosurgery as the first-line treatment of choice for trigeminal neuralgia refractory to pharmacological treatment.

## Introduction

Trigeminal neuralgia (TN) is defined as a sudden, brief, and excruciating facial pain in one or more of the branches of the trigeminal nerve (V cranial pair), leading to a severe reduction in the quality of life [1]. This craniofacial pain is a rare condition [1] that affects four to five out of every 100,000 people per year [2-4], the episodes are usually brief, but occur over weeks or months with periods of remission that may last for years [5] and the pain can be secondary to multiple causes [6].

The classical theory of the pathophysiology of TN is microvascular compression, which is due to the vascular contact usually of the superior cerebellar artery; this compression results in demyelination of the nerve fibers of the trigeminal nerve [1].

Pharmacological therapy with one or more drugs is considered the appropriate initial treatment [7]. The antiepileptic drugs carbamazepine and oxcarbazepine are the first-line pharmacological treatment for trigeminal neuralgia [1], being carbamazepine considered the most effective one [3], nevertheless, some patients do not get relief or are not well tolerated for side effects [2].

Stereotactic radiosurgery combined with pharmacological treatment is currently one of the most acceptable alternatives for the treatment of TN [8], however, this treatment is one of the most demanding radiosurgery procedures because it requires precise dose delivery [9]. Regarding the mechanism of pain relief, is thought to be focal axonal degeneration of the trigeminal nerve, affecting more pain fibers proportionally than sensory fibers. At higher doses, necrosis is more frequently observed and may contribute to the radiosurgery response [10].

Our primary endpoint was to report the short-term response (one month) outcomes of 10 patients with refractory TN after CyberKnife® (CK) radiosurgery. Secondary endpoints were to assess early side effects and complications.

## Materials and methods

Patient selection

Patients with refractory trigeminal neuralgia who underwent CyberKnife® radiosurgery between September 2022 and March 2023 at Oncoserv, Santo Domingo in the Dominican Republic participated in this study. These patients were refractory to pharmacological treatment, that is, those patients with at least six months of pharmacological management at optimal doses and who did not show a favorable response. We also selected patients who had undergone other treatments such as peripheral nerve block or decompressive microvascular surgery without favorable response, and those patients who, due to other comorbidities and age, were poor candidates for decompressive microvascular surgery and those who did not show clear evidence of such compression according to imaging studies.

The medication administered to the patients was carbamazepine alone or combined with other drugs: pregabalin+B complex in five patients, duloxetine in two patients, gabapentin in one patient, and oxycodone in one patient.

Sex, age at the time of the procedure, previous non-pharmacological treatment, region of pain, side and cranial nerve branch affected(s), and also time since diagnosis of pain were obtained from medical records. The visual analog scale (VAS), a validated and subjective measure for acute and chronic pain which values between 0 (no pain) to 10 (worst pain) [11], was used to assess facial pain before the radiosurgical procedure, as well as seven, 15 and 30 days after their treatment. Patients had a decrease in pain score greater than five units, this was considered a good and significant response [12,13].

Radiosurgical procedure - treatment planning

**Figure 1:**
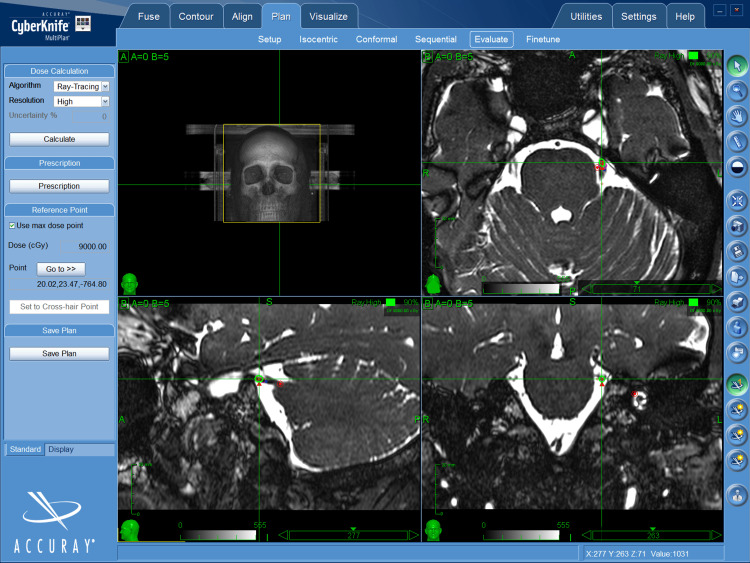
MRI fast imaging employing steady-state acquisition (FIESTA) sequence showing the trigeminal nerve at the level of the cisternal segment of the posterior fossa. The dose and treatment point was located 4-5 mm from the apparent origin of the trigeminal nerve or dorsal root entrance zone (DREZ).

**Figure 2:**
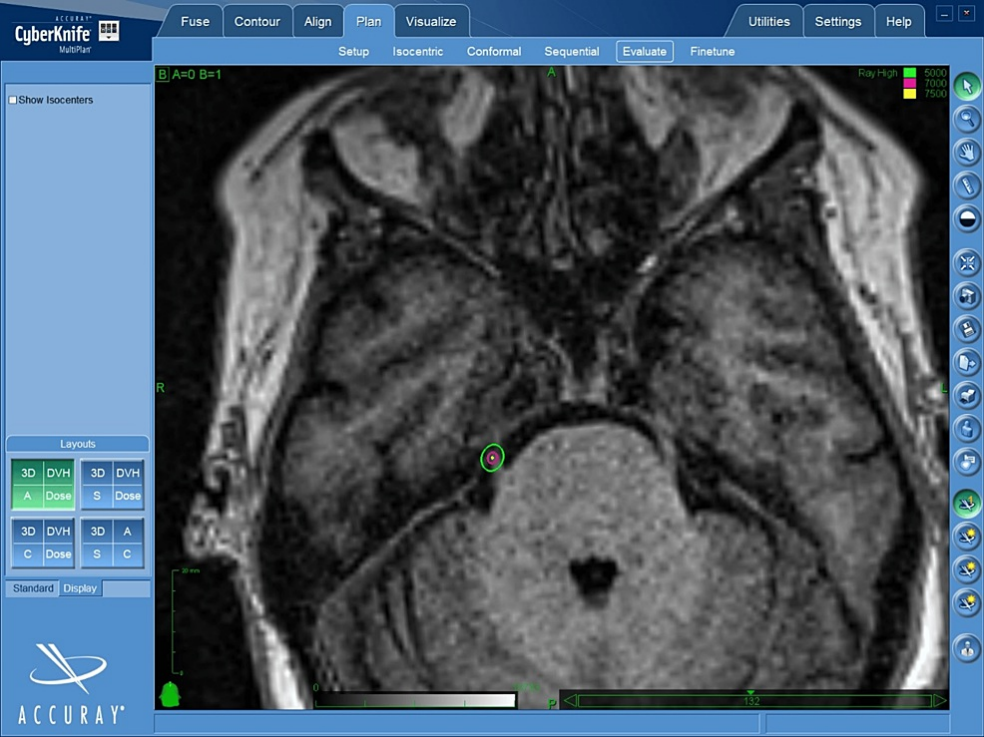
T1 MRI showing the trigeminal nerve at the level of the cisternal segment of the posterior fossa and the radiation dose prescribed for the case of a 45-year-old patient with a history of right trigeminal neuralgia in its three branches (V1, V2 and V3), previously treated with GK at a dose of 90Gy at 100%. The patient is identified as number 8 in Table 2, and has also been described previously.

The treatment point was located 4-5 mm from the apparent origin of the trigeminal nerve or dorsal root entrance zone (DREZ). The prescribed dose was 76.5 Gy at 85% at a planning target volume (PTV) of 5 mm, to achieve a dose point of 90 Gy (Figure 1), with the exception of one case of 75 Gy reirradiation (Figure 2).

The system used was CyberKnife®️ G4 radiosurgery system, which consists of a 6MV X-band linear accelerator mounted on a robotic arm that is fully articulated and has an accuracy of 0.3 mm with six degrees of freedom.

Prior to treatment, planning for each intervention included high-resolution simulation CT (1 mm slice thickness), fast imaging employing steady-state acquisition (FIESTA), 1.5 Tesla resonator MRI in T1 and T2 with and without contrast. Patients were immobilized with a standard cranial mask at CyberKnife®️. Treatment planning was then performed in MultiPlan software with CT and 3D. CyberKnife®️ treatments were developed using the 5 mm diameter cone.

Statistical analyses

The Friedman non-parametric repeated measured analysis [14] was performed to evaluate pain relief in treated patients. The resulting probability value of less than 0.05 was considered significant. Statistical analysis was performed with Jamovi [The jamovi project (2023). jamovi (version 2.3) (computer software)][15].

## Results

Table 1 shows the characteristics of 10 patients who underwent treatment, aged between 48 and 73 years, of whom all were women and 9/10 had right-sided pain. The pain interval was between three and 28 years with a mean of nine years.

**Table 1:** Characteristics of 10 patients who underwent CyberKnife treatment for refractory trigeminal neuralgia. VAS = visual analog scale.

Patient characteristics		
Age [years] mean (min-max)		58 (48-73)
Sex	Female	10 (100,0%)
	Male	0 (0,0%)
Non-pharmacological pretreatment		
	Microvascular decompression	3 (30,0%)
	Gamma Knife^®^	1 (10,0%)
	Percutaneous Trigeminal Nerve Block	3 (30,0%)
Side Pain region		
	Right hemiface	9 (90,0%)
	Left hemiface	1 (10,0%)
Affected cranial nerve branches		
	Ophthalmic (V1) and Maxilla (V2)	3 (30,0%)
	Maxilla (V2) and Mandible (V3)	6 (60,0%)
	Ophthalmic (V1), Maxilla (V2) and Mandible (V3)	1 (10,0%)
Time since pain diagnoses [years] mean (min-max)		9,7 (3-28)
Previous pain [VAS] median (min-max)		10 (10-10)
Pain at 7 days of treatment [VAS] median (min-max)		6 (4-10)
Pain at 15 days of treatment [VAS] median (min-max)		4 (2-8)
Pain at 30 days of treatment [VAS] median (min-max)		2 (0-5)

All patients had 10/10 pre-treatment pain on the VAS scale. The first month all patients had statistically significant pain response (Friedman test χ²=28.5, df=3, p<0.001). At seven days after treatment, the mean pain was 6 points, at 15 days it was 4 points and at 30 days it was 2 points.

Table 2 shows the affected branches, previous non-pharmacological treatment, pain evolution, and pain level before and after treatment with CyberKnife® for refractory trigeminal neuralgia.

**Table 2:** Branches affected, previous non-pharmacological treatment, pain evolution and pain level before and after CyberKnife® treatment for refractory trigeminal neuralgia. V1=ophthalmic branch, V2=maxilla branch, V3=mandible branch, MD=microvascular decompression, GK= Gamma Knife® radiosurgery, PTNB=percutaneous trigeminal nerve block, VAS = visual analog scale from 0 (no pain) to 10 (worst pain).

Patient	Branches affected	Previous non-pharmacologic treatment			Pain evolution (years)	Pain level (VAS)			
		MD	GK	PTNB		Before CK	7 days	15 days	30 days
1	V1 V2	Yes	No	Yes	17	10	8	8	0
2	V2 V3	No	No	No	28	10	8	4	4
3	V2 V3	No	No	No	3	10	6	2	1
4	V2 V3	No	No	No	15	10	6	4	2
5	V2 V3	No	No	No	4	10	6	4	2
6	V1 V2	No	No	No	3	10	10	6	4
7	V2 V3	No	No	No	3	10	4	2	0
8	V1 V2 V3	Yes	Yes	Yes	9	10	6	6	5
9	V2 V3	Yes	No	No	10	10	6	4	2
10	V1 V2	No	No	Yes	5	10	10	6	4

**Figure 3:**
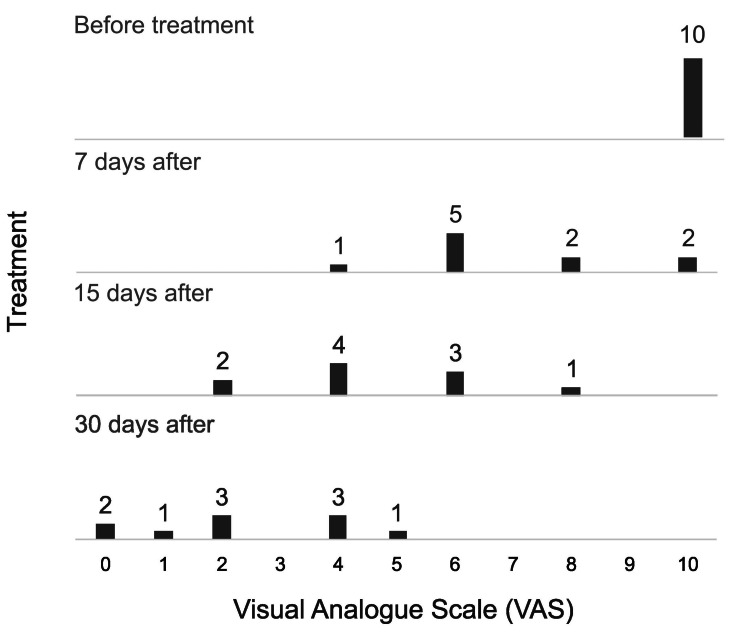
Pain level in 10 patients undergoing CyberKnife® treatment for refractory trigeminal neuralgia before and after treatment (7, 15, and 30 days after). Pain level was measured with visual analog scale (VAS) from 0 (no pain) to 10 (worst pain).

Figure 3 shows graphically the level of pain before CK treatment and at seven, 15, and 30 days after treatment. Transient facial paresthesia with resolution in less than 24 hours was present in three patients, without other complications.

## Discussion

Before treatment all patients had a pain level of 10/10 VAS; in the first week after treatment, they had an immediate response and went on to have a median level of 6/10. Immediate and successful pain relief has been reported at a median time of seven days [6,13,16]. Several series have shown that there is a direct relationship between high doses and pain response, there is a high pain-free rate using 90 Gy [17].

Romanelli et al. were the first clinical demonstration of the accuracy, safety, and efficacy of Cyberknife radiosurgery in the early relief of TN pain [16]; in this study, almost immediate pain relief (within days) was found in five of seven patients. Three of five reported subsequent pain relief at 24 hours, with a top effect at 48 hours; one patient had pain relief at 48 hours and complete pain relief at 72 hours, while another had relief between 72 hours and one week. Four of these patients had no further pain and were off the medication after six months of follow-up.

Adler et al. [18] indicate that an improvement of symptoms is experienced within a few days or weeks after radiosurgery, but the entire benefit was obtained about five months later. In the case of Tang et al. [13], they report that 11 of 14 patients showed a 5-point VAS improvement at 48-72 hours with a median prescription dose of 66 Gy. Lakshman et al. refer to their case report that immediately after treatment, the patient noticed pain relief with improvement in chewing food [6].

At 15 days, the median level was 4/10 and at 30 days this value was reduced to 2/10. One month later, two of the treated patients reported no pain at all, while the others reported the following degrees of pain: 1/10 (one patient), 2/10 (three patients), 4/10 (three patients), 5/10 (one patient). It is important to highlight that in our case of re-irradiation, the improvement of pain was 4 points at seven and 14 days (6/6 VAS), reaching a value of 5/10 VAS at 30 days.

Berti et al. [8] indicate that the largest reported series has a small group with immediate pain relief at 48 hours after treatment, but most patients have results over the initial 30 days; whereas, Romanelli et al. observed a decrease in VAS score of more than 5 points after a mean time of three weeks in 67% of patients [12].

For Gamma Knife® (GK) surgery pain relief usually begins a mean of three weeks after radiosurgery, and could continue to improve up to six months [19].

All treated patients were women, which coincides with some studies that report a higher incidence in women [20-26] but our sample is too small to be conclusive on this point.

Transient facial paresthesia with resolution in less than 24 hours was present in three patients, without other acute complications. This is consistent with Lakshman et al. [6] who indicate that their patients experienced immediate pain relief with no post-procedural complications and no significant adverse toxicities after the CyberKnife® treatment.

Antiepileptic drugs such as carbamazepine, neurontin, and phenytoin are the first line of treatment for TN [4]. During treatment, the doses received by patients were not withdrawn or changed, because abrupt discontinuation of psychotropic drugs may result in withdrawal reactions including nausea, headaches, and disturbed sleep in adults who take them [27].

In 60% of our patients, CK radiosurgery was the non-pharmacological treatment of the first choice, providing patients with satisfactory symptom relief. In our experience, even patients who received previous non-pharmacological treatments (such as microvascular decompression, GK radiosurgery, and/or percutaneous trigeminal nerve block), had a favorable short-term response with CyberKnife radiosurgery.

In this simple retrospective study, we can appreciate the usefulness of radiosurgery in the treatment of trigeminal neuralgia. CK is a tool that offers promising results with low toxicity. Patient selection is critical to obtain favorable results.

Pain relief is essential to provide quality of life for these patients, and that objective is met in this work. The results of this study coincide with those published in the literature and increasingly support this indication for treatment.

As a limitation of this research, we highlight the scarce availability of articles on short-term pain response (during the first month) in refractory trigeminal neuralgia treated with Cyberknife radiosurgery. We are evaluating the short-term results (one month) of treatment with CK, there are no articles focused as such to measure this, therefore this can serve as support for other research, however, we recognize that there is a need for studies that evaluate short-term response in larger groups with longer follow-up, we highlight that the evidence presented is clinically and statistically significant in the short term in our cases presented.

## Conclusions

High-tech CK treatment is safe, non-invasive, fast, with minimal side effects, and effective in achieving short-term pain relief in patients with refractory TN, even in those with multiple previous interventions. Given these results, we recommend evaluating CK radiosurgery as the first-line treatment of choice for trigeminal neuralgia refractory to pharmacological treatment. We are considering the need for a comparative study with a larger number of patients and a longer follow-up period.
